# The Institutional Library

**Published:** 1913-05-03

**Authors:** 


### THE INSTITUTIONAL LIBRARY.

Treatment after Operation. By William Turner, M.S., F.R.C.S., and E. R. Carling, B.S., F.R.C.S. (University of London Press: Henry Frowde and Hodder and Stoughton. 1912. Pp. 247. Price 10s. 6d. net.)

The after-treatment of operations is a very important factor in the eventual success or otherwise of the case. It is very often entrusted to those whose experience of it is not large—in hospital to the house officer, in private practice to the general practitioner; and it is very important that both these classes should have reliable text-books dealing with the thousand and one details connected with the convalescence of those who have been operated on. Hitherto there has been one manual recognised as authoritative upon this subject, that by Mr. Lockhart Mummery. Now the profession has two to choose from, and there is "very little in it" between the new book and the older one. Both are comprehensive and sound, full of practical clinical wisdom and valuable hints of all sorts. The only section in which there is any wide divergence between the two is over the important question of shock. Mr. Mummery is well known as an ardent supporter of Crile's hypotheses of the origin and treatment of this condition. Messrs. Turner and Carling do not disclose their views as to the pathology, and their treatment is a blend of the two opposing methods of Crile and Y. Henderson. Thus strychnine (worse than useless, according to one school) and pituitary extract (dangerous, according to the other) are recommended in the same breath by these authors. They also advise ten to twenty minims of ether subcutaneously as a diffusible stimulant: if they had said two drachms intramuscularly we should be more inclined to regard the procedure as of possible benefit. A device which is new to the reviewer is the instillation of a drop of suprarenal extract into the conjunctival sac for acute shock; it would be interesting to know the rationale of this, and whether it has been fully tested clinically. The authors also object to saline infusion into cellular spaces, preferring direct venous transfusion with suprarenal extract added to the salt solution. Evidently they do not share the disappointment which many surgeons now express as to the alleged virtues of suprarenal extract in shock; and the whole subject is so difficult and unsettled that divergences both of theory and practice are inevitable in connection with it. The book is a good one, but we think the price need not have exceeded six shillings or seven and six at the most, and that at half a guinea it is put beyond the reach of many who might otherwise have profited by it.

Midwifery Made Easy. By Mary L. Skinner. (London: Baillière, Tindall and Cox. 1913. Pp. 124. Price 2s. net.)

This book would appear to be a novel experiment in the written teaching of midwives, and it will be extremely interesting to watch its meed of success. The authoress seems to have borrowed the already exploited idea of a dictionary of obstetrical and gynæcological terms, and to have grafted thereupon a considerable amount of condensed obstetrical teaching. The arrangement is alphabetical, as in a dictionary, for reference, and this unfortunately entails the separation of all kinds of subjects which ought to be treated consecutively, and a corresponding excess of cross-references to remedy the defect. It will be clear from this description that the book cannot and does not take the place of an elementary text-book, which is just as much a necessity as ever; but it does supply a good deal more information than a simple dictionary can possibly do. Its chief purpose seems to be for use as a revision course just before an examination, but for such an object it contains an enormous amount of words and explanations which are quite unnecessary. The teaching and definitions are on the whole sound, though a hasty survey has revealed a few small matters which might be reconsidered for the next edition. Thus the "Obstetrical Table" for calculating the most probable date of delivery allows 280 days from the last day of the last period; whereas under "Calculation" it is stated that 280 days should be counted from the first day of the last period. We prefer the second plan, but that is a matter of opinion; Miss Skinner's views remain in doubt. The brief table concerning the artificial feeding of infants is susceptible of improvement and expansion. The total diet prescribed for the whole of the third day of life consists of three teaspoonfuls of milk and nine of water: a child thus starved and skimped would have our sympathy if his protests were vigorous and prolonged. So, too, four-hour intervals are too long for a child a month old, and the milk mixture for four months is, in our view, much too strong for most children: the quantities offered in these two cases are not stated. Under "Auscultation" we learn that the heart of a male fœtus beats faster than that of a female—a reversal of the usual teaching in this country. Under "Cord" excellent advice is given as to the method of tying, dressing, etc., but not a word as to *when* this operation should take place. Dilating bags are nowhere mentioned: though it is no part of a midwife's business to use them, we think at least she ought to know that they exist, and what they are used for. No mention is made of the fact that the essential feature of pemphigus is the presence of blebs. It is advised that a torn perinæum be sutured (by a doctor) if the tear reaches to within half an inch of the anus. We should amend this by leaving out the qualifying clause altogether: *every* torn perinæum should be seen by a doctor and sutured unless the tear is minute. The book is attractively produced, as usual with this firm of publishers, but is too bulky for the pocket or the midwifery bag.

Nisbet's Medical Directory, 1913. (London: James Nisbet and Co., Ltd.)

The chief claim to originality of this year-book is, of course, the fact that the names of the medical men are given in alphabetical order, so that this book provides probably the quickest way of getting at a practitioner's qualifications. It is, in short, the simplest of directories. Of course, something has to be paid for this virtue, as for all others, and the price is in this case brevity. Still, no doubt, the most salient facts about a medical man's technical achievements, at least educationally, are here for ready reference, and all readers, professional and lay alike, must be grateful for the handy size and the thin, but quite opaque, paper on which the letterpress is printed.

Hoblyn's Dictionary of Terms used in Medicine. By John A. P. Price, M.D.Oxon. Fifteenth Edition. Revised throughout. (London: G. Bell and Sons, Ltd. 1912. Pp. 878. Price 10s. 6d.)

This dictionary has had a long career of usefulness and still has a reputation to maintain, but we imagine that a much more severe overhauling and recasting will be necessary if it is to hold its own in the face of the competition of more recently published dictionaries. Much has been done, however, to make this fifteenth edition a worthy link in the long chain, and especially in the science of bacteriology have numerous additions been made. The expression "term used in medicine" has been interpreted widely; a large number of botanical and chemical terms is to be found, many of which might be spared with advantage. On the other hand, there are wanting several medical words—for example, acapnia—which are not uncommonly met with; and numerous most unusual expressions are given a place. The author's preface to the tenth edition, written in 1878, reads now almost pathetically; the appeals then made for etymological purity are still being made by lexicographers, but small progress has been recorded. Truly the way of the purist is hard. This dictionary possesses the merits of being most convenient in size and clear in type.

Diseases of Women. By G. E. Herman, M.B., F.R.C.S., F.R.C.P. Enlarged Edition, Revised by the Author, assisted by R. Drummond Maxwell, M.D., F.R.C.S., (London: Cassell and Co. 1913. Pp. 899. 300 Illustrations. Price 25s. net.)

Amongst the flood of new and first-class text-books on gynæcology many of the older ones have been swept away and submerged. Dr. Herman's book was too well built to be thus demolished, and in its new form it will compete vigorously with the best of the newcomers. In the three previous editions a certain peculiarity of diction had almost degenerated into a habit-spasm. The idiom is still to be found, but is now kept within bounds; it no longer detracts from, but rather adds a *sauce piquante* to, the pleasure of reading Dr. Herman's always scholarly composition. The new edition retains the symptomatic arrangement upon which the work was originally planned and executed. This classification has been abandoned by practically all recent writers in favour of a more pathological and ætiological basis. Both methods have advantages and disadvantages. Dr. Herman's scheme divorces, for instance, the treatment of ectopic gestation in its early stages from that in the (rare) later stage, when it forms an abdominal tumour: forty-two chapters, in fact, separate these two parts of what is really one indivisible subject. From the point of view of the student such a dislocation has grave drawbacks. On the other hand, a classification based upon the main symptom or symptoms complained of is very materially more helpful to the practitioner than one depending on anatomy or pathology, so that for practising doctors the method is particularly advantageous, especially in general as opposed to special practice.

Dr. Maxwell's part in the new edition has been mainly the revision and expansion of the sections dealing with surgical technique. As might be expected, the work is admirably done; and we do not think we can pay it a higher compliment than by saying it will bear comparison with Berkeley and Bonney's text-book of gynæcological surgery. Dr. Herman's opinions upon many disputed matters are as uncompromising as ever, and as forcibly argued as in previous editions. It cannot be expected that everyone will agree with all of them; but it can safely be said that the practitioner who acts up to them will soon establish a reputation in his district for this kind of work. In so encyclopædic a work omissions are hard indeed to find; nevertheless we should like to see a description in the next edition of traumatic rupture of the vagina, a case of which (with a review of the literature) is reported in the December 1912 issue of the *Middlesex Hospital Reports*. It should be added that improved binding, paper, and print bear witness to the share which the publishers have taken in this thoroughly excellent re-issue of a text-book which has long enjoyed an established reputation, and now promises to take on a new lease of useful life.

Health Through Diet. By Kenneth G. Haig, M.R.C.S., L.R.C.P. (Methuen and Co. Pp. 227. Price 3s. 6d. net.)

Dr. Alexander Haig is well known in this country as the inventor and upholder of a system of diet which he strenuously maintains is the only road to health and happiness. In the present volume his son expounds the same principles, which consist in the avoidance of all kinds of food reputed to be sources of uric acid and kindred compounds when absorbed and metabolised in the human economy. On this *index expurgatorius* are placed all meats, meat extracts, gravies, fish, fowl, and yolk of egg; all peas, beans, lentils, peanuts, and similar pulses; mushrooms, asparagus, oatmeal, brown bread, and anything containing husk of any sort; tea, coffee, cocoa, chocolate, sugar.

We are quite prepared to grant that many—nay, most—of us eat too much, and that the health of the community would be improved were a stricter moderation in diet the rule. But beyond that no important section of scientific opinion in this country supports the Haigs. As far as this book is concerned, we search it in vain for anything which can by the greatest stretch of imagination be called proof of the Uric-Acid-Free theory of diet. We find a mass of unsupported assertions, some of them demonstrably ridiculous. We find also innumerable references to other books written by the Haig family; and much adulation of Dr. Alexander Haig. Honestly, Mr. K. G. Haig's exposition is not convincing, though we do not doubt his sincerity and his belief in his father's system.

Modern Wound Treatment. By Sir G. T. Beatson, K.C.B., M.D. (Edinburgh: E. and S. Livingstone. Pp. 106. Price 2s. net.)

This small book contains a succinct outline of the Listerian principles as applied to the conduct of modern surgery. Prefixed to this account is a memoir of Lord Lister, whose portrait forms the frontispiece. The book is written in a simple and direct style, which brings it well within the capacity of any intelligent layman to comprehend. There is nothing of any striking novelty in it, in the nature of things; but as a review and résumé of current surgical teaching in the matter of wound treatment it is accurate and trustworthy.

The Medical Who's Who, 1913. (The London and Counties Press Association. Price 10s. 6d. net.)

This publication, now appearing for the second year, is evidently going to win its way in spite of the high price asked for it. Some prejudice seems originally to have existed against it owing to the idea that only selected individuals would be allowed to contribute their autobiographies-in-brief to its pages. This unfounded notion having been dissipated, the profession is showing less reluctance to supply the information necessary for the compilation of the book. This year the number of pages is double that of last year; and although it is clear that the majority of the profession even now do not take advantage of the opportunity offered them, at least there is a goodly fellowship of those who do, and one likely steadily to increase in numbers. Conducted on the discreet lines of the present management, it is not difficult to prophesy a future for this publication, which may rival in regard to the medical profession its prototype in regard to the world at large.

